# A Metabolomics Profiling Study in Hand-Foot-and-Mouth Disease and Modulated Pathways of Clinical Intervention Using Liquid Chromatography/Quadrupole Time-of-Flight Mass Spectrometry

**DOI:** 10.1155/2013/647452

**Published:** 2013-02-19

**Authors:** Cheng Lu, Xinru Liu, Xiaorong Ding, Xiao Chen, Haiwei Fan, Yunqiang Liu, Ning Xie, Yong Tan, Joshua Ko, Weidong Zhang, Aiping Lu

**Affiliations:** ^1^Institute of Basic Research in Clinical Medicine, China Academy of Chinese Medical Science, Beijing 100700, China; ^2^Department of Medicinal Chemistry of Nature Product, School of Pharmacy, Second Military Medical University, Shanghai 200433, China; ^3^Jiangxi Qingfeng Pharmaceutical Inc., Ganzhou 341000, China; ^4^School of Chinese Medicine, Hong Kong Baptist University, Kowloon Tong, Hong Kong

## Abstract

Hand-foot-and-mouth disease (HFMD), with poorly understood pathogenesis, has become a major public health threat across Asia Pacific. In order to characterize the metabolic changes of HFMD and to unravel the regulatory role of clinical intervention, we have performed a metabolomics approach in a clinical trial. In this study, metabolites profiling was performed by liquid chromatography/quadrupole time-of-flight mass spectrometry (LC-Q-TOF-MS) platform from the HFMD clinical patient samples. The outcome of this study suggested that 31 endogenous metabolites were mainly involved and showed marked perturbation in HFMD patients. In addition, combination therapy intervention showed normalized tendency in HFMD patients in differential pathway. Taken together, these results indicate that metabolomics approach can be used as a complementary tool for the detection and the study of the etiology of HFMD.

## 1. Introduction

Hand-foot-and-mouth disease (HFMD), being caused by a group of enteric viruses including enterovirus 71 (EV71), coxsackievirus A16 (CVA16), and coxsackievirus A10 (CVA10), is currently a serious epidemic in China [1]. HFMD is a common, mild, and self-limiting rash-associated illness in children under the age of 6 but could lead to mortality in large-scale outbreaks. Since first being described in California in 1969, there have been many reports on large EV71 outbreaks all over the world. Generally, severe cases of HFMD were associated with severe neurologic disorders like acute flaccid paralysis, pulmonary edema, myocarditis, and fatal encephalitis [2]. HFMD has already been emerged as an imperative global hazard, not only threatening the health of the children, but also causing tremendous loss and burden to both families and society.

Despite substantial progress that has been made in understanding many aspects of the biology and pathogenesis of HFMD, continuous search for new and more effective agents against HFMD has never been relented as the appropriate vaccines and antiviral drugs remain unavailable [3]. At the present moment, there is still no effective treatment that can work against the disease clinically [4]. Hence, Traditional Chinese Medicine (TCM) that has been proven as excellent alternative remedy against viral infection [5] could be a potential HFMD treatment option. More researches have shown that TCM plays a crucial role in improving the disease progression of HFMD over the past few decades [6]. In addition, extracts of Chinese medicines such as kappa carrageenan [3], geraniin [4], and Kalanchoe gracilis [7] have exerted strong and effective anti-EV71 and anti-CVA16 activities both *in vitro* and *in vivo*. However, since the exact pathogenesis of HFMD and the complex components of effective TCM remain unclear, it is hard to fully understand the mechanism of TCM action in the human body. Thus, the establishment of a more efficient and reliable method to evaluate the remedial effects of TCM on HFMD intervention will be of imminent importance.

The metabolomic approach has focused on studying the endogenous metabolites with low molecular weight in the biological samples. According to our knowledge, the small molecular-sized endogenous metabolites play an important role in the physiological system and represent attractive candidates for better understanding of biological phenotypes [8]. Metabolomics is a newly emerging field in analytical biochemistry other than genomics and proteomics and can be regarded as the endpoint of the “omics” cascade [9]. It aims to characterize and quantify all small metabolites that are related to drug toxicity, disease progression, environmental stimuli, and genetic variations by using nuclear magnetic resonance (NMR) spectroscopy or mass spectrometry (MS) [10]. Several studies have described the metabolomics-based research on TCM, for instance, in our laboratory when investigating pattern differentiation [11, 12] and pharmacological mechanisms [13] and in other laboratories performing quality control of Chinese herbs [14]. As metabolomics can readily detect subtle changes in the metabolic network, it is uniquely poised to increase our understanding of HFMD and the related intervention methods.

In the present study based on the liquid chromatography/quadrupole time-of-flight mass spectrometry (LC-Q-TOF-MS) platform, we investigated the serum metabolic characteristics of HFMD to gain more insight of the metabolic perturbations associated with the disease for the first time. Besides, the potential therapeutic effects and the underlying mechanisms were also explored.

## 2. Materials and Methods

### 2.1. Chemicals

LC-MS grade acetonitrile and methanol was purchased from Honeywell Burdick and Jackson (Muskegon, USA). Mass Spectroscopic grade formic acid was purchased from Fluka (Buchs, Switzerland). Distilled water was purified “in-house” using a Milli-Q20 system (Millipore; MA, USA). All metabolites standards were obtained from Sigma-Aldrich (MO, USA).

### 2.2. Sample Collection and Preparation

Eighteen severe patients with clinical symptoms of HFMD were recruited if they met the Guideline for the Diagnosis and Treatment of HFMD in China (Ministry of Health, 2010) [15] from Beijing YouAn Hospital (China) during September and October, 2010. Additional inclusion criteria were ages 1–13 years and no more than 24 hours after the occurrence of central nervous system symptoms, with any of the following: lethargy and weakness, agitation or irritability, headache, vomiting, limb weakness or acute flaccid paralysis, myoclonic jerks, ataxia, nystagmus, or oculomotor palsies. Patients who have been suffering from neurogenic pulmonary edema or heart or respiratory failure were excluded. All patients were identified as EV71-positive by a diagnostic kit (RT-PCR double Fluorescence Taqman probes, Da An Gene Company Limited, China). The patients were then assigned to combination therapy (with combination of conventional Western medicine and Chinese medicine treatments). Conventional Western medicine treatments included mannitol (0.5–1.0 g/kg) administered IV over a period of 30–40 min for every 4–8 h; glucocorticoid methylprednisolone (1-2 mg/kg/24 h); hydrocortisone (3–5 mg/kg/24 h); dexamethasone (0.2–0.5 mg/kg/24 h); intravenous immunoglobulin (IVIG; 2 g/kg) for 2–5 days. Herb derived compound Andrographolide Sulfonate (called Xiyanping injection), approved for clinical use by State Food and Drug Administration (China), is recommended for the treatment of HFMD. Xiyanping injection was administered as intravenous infusion (5–10 mg/kg/day; in 5% dextrose). The Xiyanping injection used in our study appeared as light yellow to orange clear liquid, which is composed of total andrographolide sulfonate, and manufactured by Jiangxi Qingfeng Pharmaceutical Inc. (GMP certificated). The criteria for the quality of injection procedures were in accordance with the Chinese pharmacopoeia (2010) [16]. The Xiyanping injection treatment was given in combination with conventional Western medicine cocktail for 7–10 days. The average hospital stay of these children was 7.6 days. Venous blood samples were collected in the morning preprandially from HFMD patients (before and 7 days after drug treatment) and age-matched healthy control subjects (*n* = 18). Between HFMD patient and health control group, the difference of age was not significant (*P* = 0.628, *t* test, Table 1). An Institutional Review Board had approved the previous research protocols on human subjects based on ethical and safety considerations prior to the commencement of the study and all participants had provided their informed consent in writing. One hundred *μ*L of the collected serum sample was added to 300 *μ*L methanol, and the mixture was vortexed vigorously for 30 s. After centrifugation at 9562.5 ×g for 10 min at 4°C, the supernatant was stored at −80°C until analysis [17].

### 2.3. LC-Q-TOF-MS Analysis

The LC-Q-TOF-MS analysis was performed by using an Agilent-1200 LC system, which was coupled with an electrospray ionization (ESI) source (Agilent Technologies, Palo Alto, CA, USA) and an Agilent-6520 Q-TOF mass spectrometry. The separation of all samples was performed on an Eclipse plus C18 column (1.8 *μ*m, 2.1 mm × 100 mm, Agilent) with a column temperature being set at 45°C. The flow rate was 400 *μ*L/min, and the mobile phase consisted of ultrapure water with 0.1% formic acid (A) and acetonitrile (B). The following gradient program was used: 0-1 min, 2% B; 1–3 min, 2–20% B; 3–6 min, 20–30% B; 6–11 min, 30–85% B; 11–18 min, washed with 100% B and followed by a reequilibration step of 6 min. The sample injection volume was 2 *μ*L. Mass detection was operated in both positive and negative ion modes with the following setting: drying gas (N_2_) flow rate, 8 L/min; gas temperature, 330°C; pressure of nebulizer gas, 35 psig; Vcap, 4100 V (positive mode) and 3900 V (negative mode); fragmentor, 130 V; skimmer, 65 V; scan range, *m*/*z* 80–1000. MS/MS analysis was acquired under targeted MS/MS mode with collision energy from 10 to 40 V.

### 2.4. Sequence of Analysis

The pooled QC sample was analyzed at the beginning, the end, and randomly through the analytical run to monitor the stability of sequence analysis. The typical batch sequence of serum samples consisted of the consecutive analysis of 1 QC serum sample (at the beginning of the study), followed by 6 unknown serum samples, 1 QC serum sample, before running another 6 unknown serum samples, and so forth. Meanwhile, samples were analyzed in a random order for a normal good practice. An identical sequence was repeated to complete the total set of injections (*n* = 64, including QCs) analyzed in less than 1 day per mode [18].

### 2.5. Data Analysis

The LC-MS raw data were exported and analyzed by the Agilent Mass Hunter Qualitative Analysis Software (Agilent Technologies, Palo Alto, CA, USA). Before undergoing multivariate analysis, each data was normalized by the total area to correct for the MS response shift due to the long duration between first and last injections. After this modulation, the sum of the ion peak areas within each sample was set to 10,000. Partial least squares discriminant analysis (PLS-DA) and orthogonal partial least square (OPLS) were used for analysis of metabolite profiles. Multivariate analysis was performed by the SIMCA-P software (11th version; Umetrics AB, Umeå, Sweden). Variable importance projection (VIP) was used to select differential metabolites responsible for the intergroup discrimination of each model. Statistical significance was determined by the one-way analysis of variance (ANOVA) using SPSS 13.0 for Windows (SPSS Inc., Chicago, IL, USA), followed by Duncan post hoc tests. *P* values less than 0.05 were considered significant. We obtained information about prediction of candidate metabolites corresponding with these significant *m*/*z* values by searching the biofluid metabolites database. Then, the differential RT-*m*/*z* pairs were identified between two groups by matching with tandem MS fragmentation patterns with reference standards. Relationships between differentially expressed metabolites were investigated in pathway analysis using the Ingenuity Pathway Analysis (IPA) software (Ingenuity, Redwood City, CA).

## 3. Results

### 3.1. Sample Repeatability

Extracts from six aliquots of a random blood sample were continuously injected to evaluate the repeatability. Five common extracted ion chromatograms (EICs) shared by these injections were selected according to their different chemical polarities and *m*/*z* values. The relative standard derivations (RSDs) of these peaks were 3.29%–14.54% for peak areas and 0.03%–0.96% for retention times.

### 3.2. System Stability

The LC-MS system stability for the large-scale sample analysis was demonstrated by the test of pooled QC samples. The principal components analysis (PCA) result shows that QC samples are tight clustered. Moreover, peak areas, retention times, and mass accuracies of five selected EICs in five QC samples also showed good system stability. RSDs of the five peaks were 4.74%–14.18% for peak areas, 0.02%–0.98% for retention times, and 0.10*E*−04%–0.79*E*−04% for mass accuracies. The result indicated that large-scale sample analysis had hardly any effect on the reliability of data.

### 3.3. Metabolic Comparison Based on OPLS between HFMD and Healthy Control

**Figure 1:**
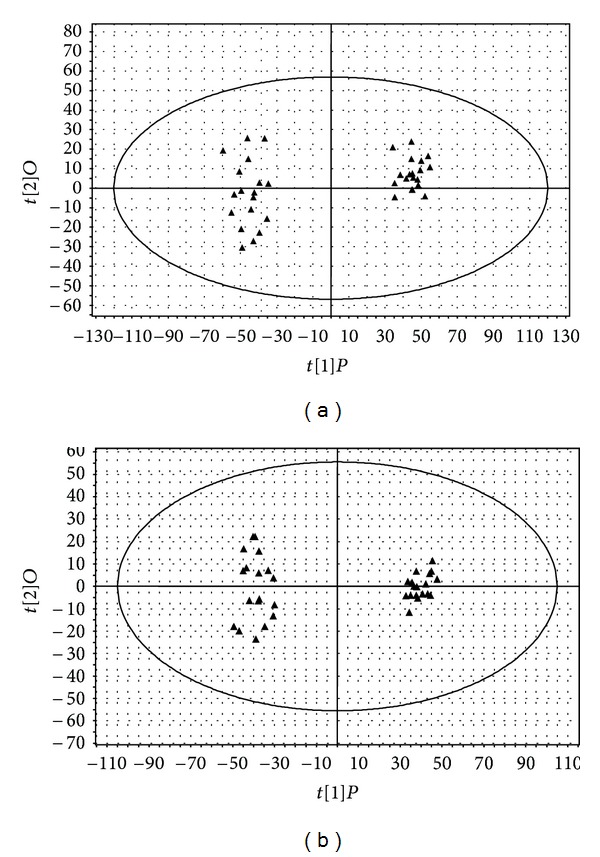
OPLS score of healthy control group and HFMD group. The score plot showed that levels of markers in serum of healthy control and HFMD patients were different. (a) Positive ion mode (*k* = 11001, *n* = 36, *R* ^2^ *Y* = 0.985, *R* ^2^ *X* = 0.321, *Q* ^2^ = 0.947, *A* = 1 + 1). (b) Negative ion mode (*k* = 11700, *n* = 36, *R* ^2^ *Y* = 0.986, *R* ^2^ *X* = 0.263, *Q* ^2^ = 0.931, *A* = 1 + 1). Each point represents an individual sample. (Left side, ▲) Samples from HFMD patients. (Right side, ▲) Samples from healthy controls.

The difference between HFMD group (patients prior to drug treatment) and healthy control group is our initial focus in this study. To maximize the difference of their metabolic profiles, a sophisticated orthogonal partial least squares projection to latent analysis (OPLS) model was applied. OPLS is a supervised method to pick out discriminating ions [19] that are contributing to the classification of samples and would remove noncorrelated variations contained within spectra [18]. Thus, OPLS was carried out to identify candidate metabolites of HFMD in this study. The OPLS models were validated in the SIMCA-P software by a default 7-round cross-validation procedure with exclusion of 1/7th of the samples from the model in each round in order to avoid the modeling overfitting caused by supervised mathematical methods [20]. The first two components were used in the OPLS model. As shown in Figures 1(a) and 1(b), there was a distinguishable classification between the clustering of HFMD group and healthy control group. *Q* ^2^ *Y* and *R* ^2^ *Y* in the OPLS models indicated that the class prediction ability of all models was high, and there was a clear difference being observed between HFMD group and healthy control group. We searched for the presumed molecular formula in the Human Metabolome Database, ChemSpider, KEGG, and some other databases to confirm possible chemical composition. According to the results, a total of the top significant 200 variables (sum of variables detected in both positive mode and negative mode) contributed to the classification of the control, and HFMD groups were selected. Furthermore, among these perturbed variables, 31 endogenous metabolites (16 in positive and 15 in negative) had been identified (Table 2).

### 3.4. The Network of Identified Metabolites between HFMD Group and Healthy Control Group

**Figure 2:**
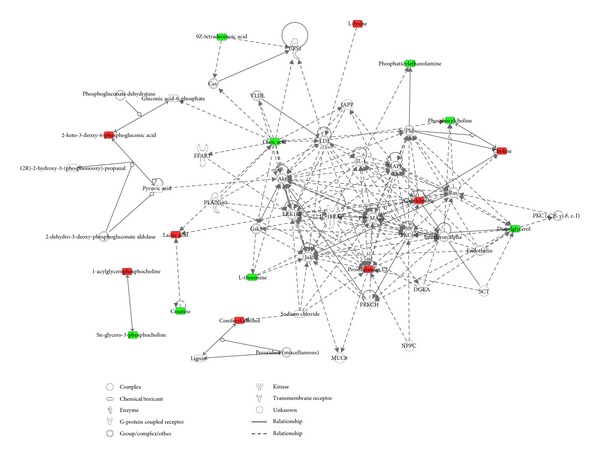
Top five significant molecular networks of HFMD related metabolites merged by IPA. Metabolites are represented as nodes, and the biological relationship between two nodes is represented as a line. Note that the colored symbols represent metabolites that occur in our data, while the transparent entries are molecules from the Ingenuity Knowledge Database. Red symbols represent upregulated metabolites in HFMD patients; green symbols represent downregulated metabolites in HFMD patients. Solid lines between molecules indicate direct physical relationship between molecules; dotted lines indicate indirect functional relationships.

Among the 31 identified metabolites, 13 were downregulated in the serum of HFMD patients, while the other 18 were upregulated (Table 2). In order to further understand the correlation between these candidate metabolites, bioinformatics analyses were performed using the IPA software, and these analyses led to the identification of biological association networks. As shown in Figure 2, it was found that most of the differential metabolites were tightly connected with each other. The established networking functions of HFMD include lipid metabolism, molecular transport, cell signaling, drug metabolism, and small molecule biochemistry, of which all play important roles in the development of HFMD. Meanwhile, the IPA software represented five top canonical pathways, including phospholipases, renin-angiotensin signaling, choline biosynthesis III, phosphatidylcholine biosynthesis I, and TREM1 signaling (Figure 2).

### 3.5. Metabolic Profiling Comparison Based on PLS among Healthy Control, HFMD, and Combination Therapy Groups

**Figure 3:**
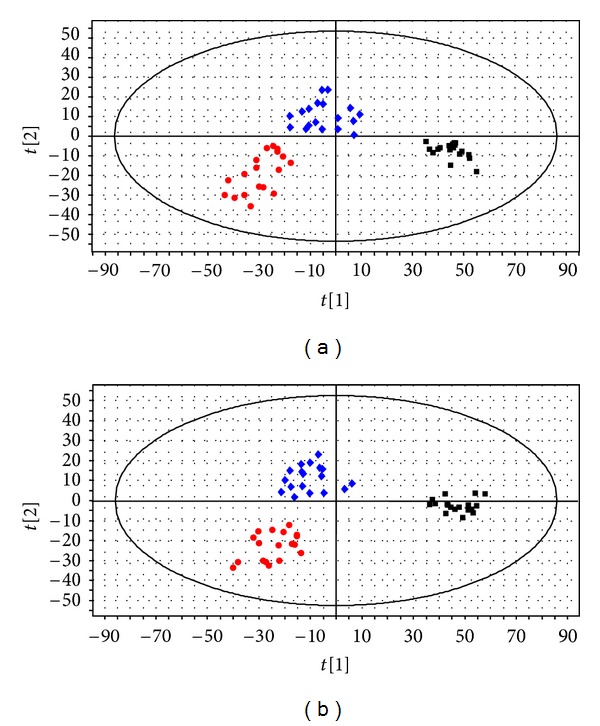
PLS-DA score plots based on the data from the healthy controls and the HFMD patients. (a) PLS-DA score plots under positive ESI ion mode. (b) PLS-DA score plots under negative ESI ion mode. The black square: healthy control group; the red circle: HFMD group; the blue diamond: combination therapy group.

In order to determine whether the serum metabolome represented in this study could be used to discriminate among different groups, partial least squares discriminant analysis (PLS-DA), a well-established supervised method that has been widely used in metabolomic study [21], was adopted so as to specify the metabolic variations associated with the disease and drug intervention. The first two components were used in the PLS-DA model. As shown in Figure 3, there was a distinguishable classification between the clustering of HFMD group, CT group (patients 7 days after combination therapy), and control group. Figures 3(a) and 3(b) displayed that separation of the groups could be achieved with the model parameters *R* ^2^ *Y* = 0.875, *Q* ^2^ = 0.710 for positive ESI ion mode and *R* ^2^ *Y* = 0.908, *Q* ^2^ = 0.727 for negative ESI ion mode. *Q* ^2^ *Y* obtained from cross-validation procedure represents the predictive accuracy of the model, and *R* ^2^ *Y* shows how well the model fits to the data. The previous parameters indicated that the two models had good ability to explain the data. Moreover, the results from permutation tests also showed that the two models were not over-fitting and were efficient and reliable (intercepts: *R* ^2^ = 0.167, *Q* ^2^ = −0.172 for positive ion mode data and *R* ^2^ = 0.541, *Q* ^2^ = −0.209 for negative ion mode data). We therefore conclude that HFMD had altered the serum metabolic profiles of patients when compared with healthy individuals, with the perturbations occurring in CT group.

### 3.6. The Evaluation of Combination Therapy

**Figure 4:**
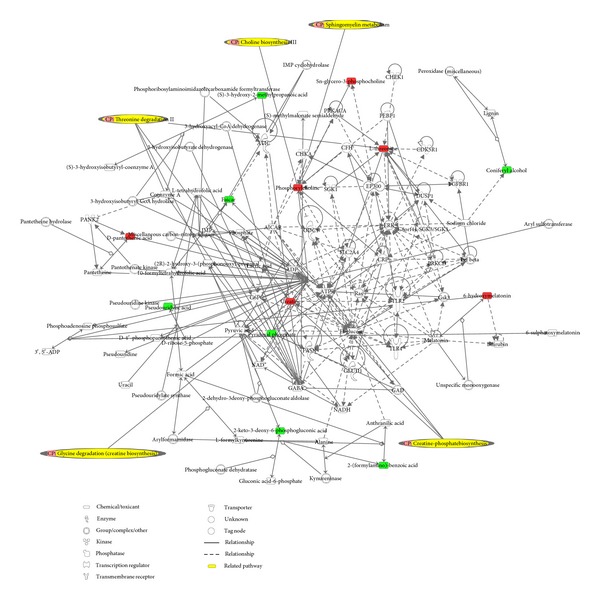
Significant molecular network of combination therapy related metabolites. Metabolites are represented as nodes, and the biological relationship between two nodes is represented as a line. Note that the colored symbols represent metabolites that occur in our data, while the transparent entries are molecules from the Ingenuity Knowledge Database. Red symbols represent up-regulated metabolites in HFMD patients after combination therapy; green symbols represent down-regulated metabolites in HFMD patients after combination therapy. Solid lines between molecules indicate direct physical relationship between molecules; dotted lines indicate indirect functional relationships. The yellow dashed area denotes the related pathway.

By using a similar method, we have identified the significant variables (sum of variables being detected in both positive mode and negative mode) based on the LC-Q-TOF-MS platform, which could contribute to the classification among the groups. As shown in Table 2, the metabolites with dashed area (15 metabolites) were modulated to the normal level in CT group, indicating the potential treatment targets of combination therapy. Associated network functions represented by IPA software include amino acid metabolism, molecular transport, and small molecule biochemistry. IPA also performed five top canonical pathways of these metabolites, including creatine-phosphate biosynthesis, sphingomyelin metabolism, glycine degradation, threonine degradation II, and choline biosynthesis III (Figure 4).

## 4. Discussion

Human EV71-associated HFMD has become a leading cause of childhood infection in China since 2008. Epidemic and molecular characteristics of HFMD have been examined in many areas of the country [2]. However, clinical and metabolomic characterization of HFMD remains scarce. Metabolomics approach can reveal the profile of endogenous metabolites of low molecular weight in biofluids that are related to disease progression, since it seeks to discover and capitalize on the metabolic derangements that occurred in the body as a result of the mutated genotype being manifested before actual gross phenotypic changes [22]. Many metabolomic efforts have been made to investigate the biomarkers and efficacy evaluation in viral diseases, such as infections by hepatitis C virus (HCV) [23], influenza A virus [24], and HIV-1 [25]. In the present study, the application of metabolomics is well suited for exploration of HFMD.

The compendium that we generated on the changes during the disease processes of HFMD has highlighted a wide variety of molecular and metabolic alterations. More detailed analyses of networks and pathways bring influenced were performed by IPA software. Our data have suggested lipid metabolism as the first important network function of perturbation in HFMD group. From the differentiating metabolites in HFMD, evident alteration in lipid metabolism was observed in HFMD patients following EV71 infection. This finding in fact echoes with another similar work at the proteomic level [26]. Lipid metabolism can be affected in a variety of ways during viral infection, whereas the major lipid changes involve triglycerides, free fatty acids, and ketone bodies, and so on [27]. Studies have demonstrated the relationship between HIV [28], HCV [29–31], and lipid metabolism, with evaluation of the role of interventions during dyslipidemia. Concomitantly, IPA had generated three related pathways, namely, phospholipases, choline biosynthesis III, and phosphatidylcholine biosynthesis I, which provides direct evidence in the perturbation of lipid metabolism in HFMD. These interrelated pathways implicate that lipid metabolism may be influenced by virus infection. Furthermore, we mainly focused on evaluating individual candidate metabolites during the analysis of lipid metabolism. We observed significantly lower levels of diacylglycerol (DAG) in HFMD patients when compared with those in the healthy control group. DAG is a versatile molecule that participates as substrate in the synthesis of structural and energetic lipids. DAG in lipid droplets from cytoplasm liver cells is associated with insulin resistance in nondiabetic obese human [32]. The termination of DAG signaling by DAG kinases can lead to inhibition of antiviral cytokine production. HIV-1 virus gene expression can be induced by DAG synthesized on a five-member ring platform [33]. This finding might well supplement the pathogenesis of HFMD. Nonetheless, phosphatidylethanolamine (PE) level was found to be decreased in HFMD patients. As a major component in the mammalian plasma membrane, PE is mainly present in the inner leaflet of the membrane bilayer in viable and typical mammalian cells. PE is likely to be a versatile chemical species that plays certain role in the regulation of defined biological and physiological activities [34]. Choline, the basic constituent of lecithin that is found in many plants and animal organs, is up-regulated in HFMD patients. It is essential as a precursor of acetylcholine, as a methyl donor in various metabolic processes, and also in lipid metabolism [35]. Nevertheless, alterations of the cerebral choline can be observed in HCV-infection patients [36, 37]. Altogether, the altered lipid metabolite may be a sign of HFMD and reflect the abnormal lipid metabolism status in HFMD patients. Our results could provide a hint for further understanding of the role of lipid metabolism in the pathogenesis of HFMD.

From the signal transduction perspective, renin-angiotensin signaling is closely related. It is well known that renin-angiotensin proteins are key regulators of other signaling cascades, controlling many biological processes such as proliferation, differentiation, and apoptosis [38]. Renin-angiotensin signaling could be potential drug targets to determine the appropriate therapeutic intervention of HFMD. Nevertheless, additional prospective work is needed to investigate the precise mechanism.

It is of interest to note that inflammatory reactions were found to be dysregulated in HFMD patients. It has been considered that inflammation plays an important role during the HFMD process. The milder HFMD cases presented systemic inflammatory response syndrome status in clinical trials [39]. Moreover, patients with central nervous system diseases were found to have acute inflammation of the grey matter at the brain stem [40]. In a basic research, investigators had identified that human P-selectin glycoprotein ligand-1 (PSGL-1; CD162), a sialomucin membrane protein expressed on leukocytes, may play a major role in early stages of inflammation by interacting with selections and chemokines, as a functional receptor for EV71 [41]. Another report also indicated that massive induction of proinflammatory cytokines is responsible for the pathogenicity of EV71 [42]. Urocanic is a breakdown (deamination) product of histidine. In the liver, urocanic acid (UCA) is an intermediate in the conversion of histidine to glutamic acid, whereas in the epidermis, it accumulates and may be both a UV protectant and an immunoregulator [43]. *cis*-UCA can increase cytokine protein production including that of TNF-alpha, IL-6, and IL-8 in a dose-dependent manner [44]. Prostaglandin E_2_ (PGE_2_) is the most common and biologically active prostanoid among mammalian prostaglandins, which has been traditionally regarded as a mediator of immune inflammation [45]. It is interesting that PG-endoperoxide synthase-2 is dramatically induced by *cis*-UCA, resulting in an enhanced secretion of PGE_2_. A relatively high level of UCA, together with PGE_2_, was concurrently found in the HFMD group. These results suggest that the inflammation-related processes in HFMD may be largely perturbed. 

HFMD has multiple alternative pathogenic pathways that lead to a particular pathophysiological state, with a wide range of resulting phenotypes. Thus, it provides a significant treatment venture in clinical practice. In this study, we performed combination therapy to determine metabolic changes in HFMD patients. Among 31 HFMD-related metabolites, 15 metabolites were reversed to the level of the health control group, suggesting that the combination therapy has a positive impact on repairing the abnormal metabolic profiles in HFMD. In CT group, the altered pathways include amino acid metabolism (threonine degradation II and glycine degradation) and lipid metabolism (creatine-phosphate biosynthesis, sphingomyelin metabolism, and choline biosynthesis III) (Figure 4). Amino acid metabolism, especially that of threonine degradation II, glycine degradation being addressed by IPA, is involved in the modulation process of combination therapy based on results from this study. In agreement with previous observation, the amino acid metabolism in patients with hepatitis B virus (HBV) infection is significantly changed [46]. Conserved glycine 33 residue in flexible domain I of HCV core protein is critical for its virulent activity [47]. A previous study has demonstrated that HBV E-antigen physically associates with receptor interaction serine/threonine protein kinase 2 [48]. As a result, our data are consistent with the phenomenon on the interference of glycine and threonine metabolism in viral diseases. We therefore believe that the primary regulatory targets of combination therapy involve modulation of amino acid metabolism. Interestingly, the related pathway of lipid metabolism again showed important role in the regulation of HFMD in CT group, consistent well with the before mentioned. Manipulation of these pathways could aim at the prime targets in successful HFMD therapy in CT group. 

## 5. Conclusions

In summary, a metabolomic approach based on LC-Q-TOF-MS platform was employed to study the metabolic characteristics between HFMD patients and healthy individuals, as well as combination therapy interventions. A clear separation between HFMD patients and healthy subjects was achieved. As a result, 31 endogenous metabolites showed marked perturbation in HFMD patients. We also observed a number of important pathway perturbations in HFMD patients in association with lipid metabolism and inflammation reaction, and so forth. Combination therapy shows positive modulation in HFMD patients in different pathway. Metabolomic technique was deemed useful in the exploration of the complex metabolic states of HFMD, which can also provide a theoretical basis for the prevention and treatment of the disease. 

## Figures and Tables

**Table 1:** Basic feature of HFMD group and healthy control group.

Group	Number (*n*)	Gender (*n*)		Age (month)^a^
		Male	Female
Healthy control	18	13	5	27.1 ± 10.9
HFMD	18	11	7	28.9 ± 11.6

^a^Data are expressed as mean ± SD.

**Table 2:** Identified markers between healthy control and HFMD.

Mode	R.T (min)	Exact mass	Formula	Compound (Resource)	Significance/fold change		
					G2 versus G1	G3 versus G2	G3 versus G1
	1.8	145.1103	C_7_H_15_NO_2_	Acetylcholine (HMDB)	**/ 1.48	**/ 0.65	**/ 0.77
	2.5	202.1205	C_10_H_18_O_4_	Sebacic acid (HMDB)	**/ 1.58	**/ 0.67	N.S
	2.6	90.0317	C_3_H_6_O_3_	Lactic acid (HMDB)	**/ 13.4	N.S	**/ 13.4
	3.6	258.0141	C_6_H_11_O_9_P	2-Keto-3-deoxy-6-phosphogluconic acid (HMDB)	**/ 5.55	**/ 0.12	N.S
	3.7	247.0246	C_8_H_10_NO_6_P	Pyridoxal phosphate (HMDB)	**/ 5.23	**/ 0.31	N.S
	6.0	219.1107	C_9_H_17_NO_5_	D-Pantothenic acid (HMDB)	**/ 0.47	**/ 2.28	N.S
	6.2	138.0429	C_6_H_6_N_2_O_2_	Urocanic acid (HMDB)	**/ ∞	N.S	**/ ∞
ESI (+)	10.1	180.0634	C_6_H_12_O_6_	beta-D-Glucose (HMDB)	**/ 0.38	N.S	**/ 0.37
	13.7	519.3325	C_26_H_50_NO_7_P	1-18:2(9Z,12Z) Lysophosphatidylcholine (HMDB)	**/ 0.49	**/ 1.84	N.S
	12.71	169.0504	C_4_H_12_NO_4_P	Phosphorylcholine (HMDB)	**/ 0.13	**/ 5.98	N.S
	12.72	250.1205	C_14_H_18_O_4_	Ubiquinone (HMDB)	**/ 0.27	**/ 0.22	**/ 0.06
	14.1	671.489	C_37_H_70_NO_7_P	Phosphatidylethanolamine (KEGG)	**/ 0	N.S	**/ 0
	14.3	714.5223	C_47_H_70_O_5_	Diacylglycerol (HMDB)	**/ 0	N.S	**/ 0
	14.7	571.3638	C_30_H_54_NO_7_P	1-Acyl-sn-glycerol-3-phosphocholine (HMDB)	**/ 2.02	*/ 0.88	*/ 1.78
	16.4	103.0997	C_5_H_13_NO	Choline (HMDB)	**/ 1.69	**/ 0.69	*/ 1.17
	17.8	522.3604	C_28_H_50_N_4_O_3_S	Oleic acid (HMDB)	**/ 0	N.S	**/ 0

	1.5	146.1055	C_6_H_14_N_2_O_2_	L-Lysine (HMDB)	**/ 1.56	**/ 1.48	**/ 2.02
	1.6	336.0484	C_11_H_15_NO_9_P	Nicotinate D-ribonucleotide (HMDB)	**/ 2.58	N.S	**/ 2.73
	1.8	131.0695	C_4_H_9_N_3_O_2_	Creatine (HMDB)	**/ 0.43	**/ 2.08	N.S
	2.1	119.0582	C_4_H_9_NO_3_	L-Threonine (HMDB)	**/ 0.46	**/ 2.07	N.S
	4.28	366.0577	C_10_H_15_N_4_O_9_P	FAICAR (ChemSpider)	**/ 16.24	**/ 0.04	N.S
	4.29	104.0473	C_4_H_8_O_3_	(S)-3-Hydroxy-2-methylpropanoic acid (HMDB)	**/ 1.77	**/ 0.54	N.S
	5.4	165.0426	C_8_H_7_NO_3_	2-(Formylamino)-benzoic acid (HMDB)	*/ 2.08	**/ 0.35	N.S
	5.6	180.0786	C_10_H_12_O_3_	Coniferyl alcohol (HMDB)	**/ ∞	**/ 0	N.S
ESI (−)	6.5	258.1106	C_8_H_21_NO_6_P	sn-glycero-3-Phosphocholine (HMDB)	**/ 0.26	*/ 7.93	N.S
	8.0	248.1161	C_13_H_16_N_2_O_3_	6-Hydroxymelatonin (ChemSpider)	**/ 0.18	*/ 4.25	N.S
	10.0	276.1321	C_11_H_20_N_2_O_6_	Saccharopine (HMDB)	**/ ∞	**/ 0.24	**/ ∞
	10.8	324.0359	C_9_H_13_N_2_O_9_P	Pseudouridylic acid (ChemSpider)	**/ ∞	**/ 0	N.S
	12.8	308.041	C_9_H_13_N_2_O_8_P	dUMP (HMDB)	**/ 26.7	**/ 0.1	*/ 46.8
	15.0	364.2613	C_22_H_36_O_4_	Prostaglandin E2 (HMDB)	**/ 2.10	N.S	**/ 1.83
	16.6	226.1933	C_14_H_26_O_2_	9Z-Tetradecenoic acid (HMDB)	**/ 0.49	N.S	**/ 0.47

G1: healthy control group; G2: HFMD group; G3: combination therapy group; HMDB: Human Metabolome Database; KEGG: Kyoto Encyclopedia of Genes and Genomes. (*∞*: represented denominator is zero;  *P < 0.05;  **P < 0.01; N.S: no significance).

## Abbreviations

HFMD: Hand-foot-and-mouth disease
EV71: Enteric viruses including enterovirus 71
CVA16: Coxsackievirus A16
CVA10: Coxsackievirus A10
TCM: Traditional Chinese Medicine
LC-Q-TOF-MS: Liquid chromatography/quadrupole time-of-flight mass spectrometry
ESI: Electrospray ionization.

## References

[1] Chen G, Lu C, Zha Q (2012). A network-based analysis of traditional Chinese medicine cold and hot patterns in rheumatoid arthritis. *Complementary Therapies in Medicine*.

[2] Wang X, Zhu C, Bao W (2012). Characterization of full-length enterovirus 71 strains from severe and mild disease patients in northeastern China. *PLoS ONE*.

[3] Chiu Y-H, Chan Y-L, Tsai L-W, Li T-L, Wu C-J (2012). Prevention of human enterovirus 71 infection by kappa carrageenan. *Antiviral Research*.

[4] Yang Y, Zhang L, Fan X, Qin C, Liu J (2012). Antiviral effect of geraniin on human enterovirus 71 in vitro and in vivo. *Bioorganic and Medicinal Chemistry Letters*.

[5] Wang C, Cao B, Liu Q-Q (2011). Oseltamivir compared with the Chinese traditional therapy maxingshigan-yinqiaosan in the treatment of H1N1 influenza: a randomized trial. *Annals of Internal Medicine*.

[6] Xue B, Yao Z, Yu R (2011). Studies on anti-EV71 virus activity of traditional Chinese medicine and its clinical application in treatment of HFMD. *Zhongguo Zhongyao Zazhi*.

[7] Wang C-Y, Huang S-C, Zhang Y (2012). Antiviral ability of Kalanchoe gracilis leaf extract against Enterovirus 71 and coxsackievirus A16. *Evidence-based Complementary and Alternative Medicine*.

[8] Allen AE, Dupont CL, Oborník M (2011). Evolution and metabolic significance of the urea cycle in photosynthetic diatoms. *Nature*.

[9] Dettmer K, Hammock BD (2004). Metabolomics—a new exciting field within the “omics” sciences. *Environmental Health Perspectives*.

[10] Maher AD, Lindon JC, Nicholson JK (2009). H NMR-based metabonomics for investigating diabetes. *Future Medicinal Chemistry*.

[11] Van Wietmarschen H, Yuan K, Lu C (2009). Systems biology guided by Chinese medicine reveals new markers for sub-typing rheumatoid arthritis patients. *Journal of Clinical Rheumatology*.

[12] Gu Y, Lu C, Zha Q (2012). Plasma metabonomics study of rheumatoid arthritis and its Chinese medicine subtypes by using liquid chromatography and gas chromatography coupled with mass spectrometry. *Molecular BioSystems*.

[13] Zhao H, Li J, He X (2012). The protective effect of Yi Shen Juan Bi Pill in arthritic rats with castration-induced kidney deficiency. *Evidence-based Complementary and Alternative Medicine*.

[14] Dong H, Zhang A, Sun H (2012). Ingenuity pathways analysis of urine metabolomics phenotypes toxicity of Chuanwu in Wistar rats by UPLC-Q-TOF-HDMS coupled with pattern recognition methods. *Molecular BioSystems*.

[15] (2010). *The Ministry of Health Guideline for the Diagnosis and Treatment of Hand Foot and Mouth Disease*.

[16] (2010). *Pharmacopoeia Committee of People's Republic of China: Chinese Pharmacopoeia*.

[17] Dunn WB, Broadhurst D, Begley P (2011). Procedures for large-scale metabolic profiling of serum and plasma using gas chromatography and liquid chromatography coupled to mass spectrometry. *Nature Protocols*.

[18] Lv Y, Liu X, Yan S (2010). Metabolomic study of myocardial ischemia and intervention effects of Compound Danshen Tablets in rats using ultra-performance liquid chromatography/quadrupole time-of-flight mass spectrometry. *Journal of Pharmaceutical and Biomedical Analysis*.

[19] Tapp HS, Kemsley EK (2009). Notes on the practical utility of OPLS. *TrAC—Trends in Analytical Chemistry*.

[20] Jiang P, Dai W, Yan S (2011). Potential biomarkers in the urine of myocardial infarction rats: a metabolomic method and its application. *Molecular BioSystems*.

[21] Williams RE, Lenz EM, Evans JA (2005). A combined 1H NMR and HPLC-MS-based metabonomic study of urine from obese (fa/fa) Zucker and normal Wistar-derived rats. *Journal of Pharmaceutical and Biomedical Analysis*.

[22] Taylor SL, Ganti S, Bukanov NO (2010). A metabolomics approach using juvenile cystic mice to identify urinary biomarkers and altered pathways in polycystic kidney disease. *American Journal of Physiology*.

[23] Godoy MMG, Lopes EPA, Silva RO (2010). Hepatitis C virus infection diagnosis using metabonomics. *Journal of Viral Hepatitis*.

[24] Lin S, Liu N, Yang Z (2010). GC/MS-based metabolomics reveals fatty acid biosynthesis and cholesterol metabolism in cell lines infected with influenza A virus. *Talanta*.

[25] Maher AD, Cysique LA, Brew BJ, Rae CD (2011). Statistical integration of 1H NMR and MRS data from different biofluids and tissues enhances recovery of biological information from individuals with HIV-1 infection. *Journal of Proteome Research*.

[26] Deng L, Jia HL, Liu CW (2012). Analysis of differentially expressed proteins involved in hand, foot and mouth disease and normal sera. *Clinical Microbiology and Infection*.

[27] Blackburn GL (1977). Lipid metabolism in infection. *American Journal of Clinical Nutrition*.

[28] Dubé MP, Cadden JJ (2011). Lipid metabolism in treated HIV infection. *Best Practice and Research: Clinical Endocrinology and Metabolism*.

[29] Koike K, Tsutsumi T, Yotsuyanagi H, Moriya K (2010). Lipid metabolism and liver disease in hepatitis C viral infection. *Oncology*.

[30] Negro F (2010). Abnormalities of lipid metabolism in hepatitis C virus infection. *Gut*.

[31] Targett-Adams P, Boulant S, Douglas MW, McLauchlan J (2010). Lipid metabolism and HCV infection. *Viruses*.

[32] Kumashiro N, Erion DM, Zhang D (2011). Cellular mechanism of insulin resistance in nonalcoholic fatty liver disease. *Proceedings of the National Academy of Sciences of the United States of America*.

[33] Hamer DH, Bocklandt S, McHugh L (2003). Rational design of drugs that induce human immunodeficiency virus replication. *Journal of Virology*.

[34] Zhao M (2011). Lantibiotics as probes for phosphatidylethanolamine. *Amino Acids*.

[35] http://www.hmdb.ca/metabolites/HMDB00097.

[36] Weissenborn K, Tryc AB, Heeren M (2009). Hepatitis C virus infection and the brain. *Metabolic Brain Disease*.

[37] Forton DM, Allsop JM, Cox IJ (2005). A review of cognitive impairment and cerebral metabolite abnormalities in patients with hepatitis C infection. *AIDS*.

[38] Castellano E, Downward J (2010). Role of RAS in the regulation of PI 3-kinase. *Current topics in Microbiology and Immunology*.

[39] Fu D, Li CR, He YX (2009). Changes of immune function in patients with enterovirus 71 infection. *Chinese Journal of Pediatrics*.

[40] Koroleva GA, Lukashev AN, Khudyakova LV, Mustafina AN, Lashkevich VA (2010). Encephalomyelitis caused by enterovirus type 71 in children. *Voprosy Virusologii*.

[41] Nishimura Y, Shimojima M, Tano Y, Miyamura T, Wakita T, Shimizu H (2009). Human P-selectin glycoprotein ligand-1 is a functional receptor for enterovirus 71. *Nature Medicine*.

[42] Khong WX, Foo DGW, Trasti SL, Tan EL, Alonso S (2011). Sustained high levels of interleukin-6 contribute to the pathogenesis of enterovirus 71 in a neonate mouse model. *Journal of Virology*.

[43] http://www.hmdb.ca/metabolites/HMDB00301.

[44] Kaneko K, Smetana-Just U, Matsui M (2008). Cis-urocanic acid initiates gene transcription in primary human keratinocytes. *Journal of Immunology*.

[45] Sakata D, Yao C, Narumiya S (2010). Prostaglandin E2, an immunoactivator. *Journal of Pharmacological Sciences*.

[46] Castorena KM, Stapleford KA, Miller DJ (2010). Complementary transcriptomic, lipidomic, and targeted functional genetic analyses in cultured *Drosophila* cells highlight the role of glycerophospholipid metabolism in Flock House virus RNA replication. *BMC Genomics*.

[47] Angus AGN, Loquet A, Stack SJ (2012). Conserved glycine 33 residue in flexible domain I of hepatitis C virus core protein is critical for virus infectivity. *Journal of Virology*.

[48] Wu S, Kanda T, Imazeki F (2012). Hepatitis B virus e antigen physically associates with receptor-interacting serine/threonine protein kinase 2 and regulates IL-6 gene expression. *Journal of Infectious Diseases*.

